# Adult Arabs have higher risk for diabetes mellitus than Jews in Israel

**DOI:** 10.1371/journal.pone.0176661

**Published:** 2017-05-08

**Authors:** Anat Jaffe, Shmuel Giveon, Liat Wulffhart, Bernice Oberman, Maslama Baidousi, Arnona Ziv, Ofra Kalter-Leibovici

**Affiliations:** 1 Endocrinology and Diabetes Unit, Hillel Yaffe Medical Center, Hadera, Israel; 2 Clalit Health Services, Tel Aviv, Israel; 3 Department of Family Medicine, Tel-Aviv University, Tel Aviv, Israel; 4 Unit of Biostatistics; Gertner Institute for Epidemiology & Health Policy Research, Sheba Medical Center, Tel-Hashomer, Ramat Gan, Israel; 5 Information and Computer Unit, Gertner Institute for Epidemiology & Health Policy Research, Sheba Medical Center, Tel-Hashomer, Ramat Gan, Israel; 6 Unit of Cardiovascular Epidemiology, Gertner Institute for Epidemiology & Health Policy Research, Sheba Medical Center, Tel-Hashomer, Ramat Gan, Israel; 7 Sackler Faculty of Medicine, Tel-Aviv University, Tel-Aviv, Israel; University of Oxford, UNITED KINGDOM

## Abstract

**Objective:**

Diabetes mellitus is an emerging epidemic in the Arab world. Although high diabetes prevalence is documented in Israeli Arabs, information from cohort studies is scant.

**Methods:**

This is a population study, based on information derived between 2007–2011, from the electronic database of the largest health fund in Israel, among Arabs and Jews. Prevalence, 4-year-incidence and diabetes hazard ratios [HRs], adjusted for sex and the metabolic-syndrome [MetS]-components, were determined in 3 age groups (<50 years, 50–59 years, and ≥60 years).

**Results:**

The study cohort included 17,044 Arabs (males: 49%, age: 39.4±17.3) and 16,012 Jews (males: 50%, age: 40.5 ±17.6). The overall age and sex-adjusted diabetes prevalence rates were much higher among Arabs 18.4% (95%CI: 17.6–19.1); and 10.3% (95%CI: 9.7–10.9) among Jews. Arab females had higher prevalence rates 20.0% (95%CI: 19–21) than Arab males 16.7% (95%CI: 15.7–17.8). Annual incidence rates were also significantly higher among Arabs 2.9% (95%CI: 2.7–3.1) than among Jews 1.7% (95%CI: 1.6–1.8). This held true across all age and sex subgroups. Adjustment for body mass index [BMI] attenuated HR estimates associated with Arab ethnicity across all age subgroups, mainly in the <50yrs age group from HR 2.04 (95%CI: 1.74–2.40) to 1.64 (95%CI: 1.40–1.92). BMI at incident diabetes among females was higher in Arabs than Jews. Males, however, did not differ by ethnicity.

**Conclusion:**

Arabs, mainly female, have high incidence and prevalence of diabetes. This excess risk is only partially explained by the high prevalence of obesity. Effective culturally-congruent diabetes prevention and treatment and an effective engagement partnership with the Arab community are of paramount need.

## Introduction

Arabs, mainly Muslim (82%), are the largest ethnic minority group in Israel, accounting for 20.7% of the total Israeli population [1]. Most of the Arab population in Israel lives in predominantly Arab communities in close proximity to Jewish towns, with high exposure to westernized foods and eating habits, while the rest live in mixed Jewish/Arab cities [2]. The Arab communities are still undergoing a transition from a mainly agrarian society, to a more urbanized one. On the whole, compared to the Jewish population, Arabs in Israel have lower socioeconomic status and poorer health awareness [3].

The accelerated urbanization has been accompanied by a nutrition transition, resulting in lower levels of physical activity, and the exchange of traditional foods high in complex carbohydrates for new foods high in refined carbohydrates [4, 5]. These changes explain the high prevalence of obesity among Arabs [6].

In a population-based cross-sectional survey among 1,100 adult Jewish and Arab Israelis, the prevalence of diabetes was higher among Arab participants than among Jewish participants (21.0% vs. 11.9%), respectively. Diabetes risk calculation was based on self-reported age at diabetes diagnosis [7]. The excess diabetes risk among Arabs was independent of body mass index [BMI], family history of diabetes, and consumption of energy-dense foods [7]. Although prevalence data are crucial for appreciation of disease burden, they reflect both disease risk and survival. Indeed, diabetes-related mortality accounts for a significant part of the disparity in longevity among Arabs and Jews in Israel [8].

The objectives of this study were to determine the prevalence and incidence rates of diabetes in a large cohort of Arabs and Jews living in Israel, based on data from a largest health fund in Israel. Diabetes risk was also studied after adjustment for the individual components of the metabolic syndrome (MetS).

## Subjects and methods

This was a historical-prospective cohort study. The study cohort included residents of the mostly urban Sharon-Shomron area in central Israel who were insured by Clalit Health Services (CHS), the largest health fund in Israel. CHS insures 76% of the district’s Arab population and 46% of the Jewish population. Patient-related information is continually recorded in patient electronic medical records and in the CHS administrative database. The database includes a list of all diagnoses, demographic information, laboratory tests and imaging results, chronic medical therapy and hospital admissions. This database was the sole source for the study data.

Jewish and Arab Israelis, matched for sex and age group, and who were 20 years or older on 12.31.2007 and survived until at least 1.1.2009, were included in the sample. The Jewish Israeli sample did not include Ethiopian Jews who are a unique ethnic group with a high prevalence of diabetes, accounting for less than 2% of the Jewish population in Israel [9].

We used data collected from 01.01.2007 through 12.31.2011. Prevalent diabetes was defined as diagnosis of diabetes or purchases of 3 or more hypoglycemic drug prescriptions between 01.01.2007 and 12.31.2007. Incident diabetes was defined as a physician diagnosis of diabetes, purchases of 3 or more hypoglycemic drug prescriptions, or at least two raised values tested within a period of 12 months of: either a fasting glucose (≥126mg/dl) or a post-75gr oral glucose load 2-hour plasma test (≥200mg/dl) or a HbA1c (≥6.5%, i.e. ≥48 mmol/mol) [10], between 01.01.2008 and 12.31.2011, among participants who were not diagnosed as diabetic prior to 12.31.2007.

### Ethical considerations

The CHS ethics committee approved the study protocol. In accordance with the Israeli Ministry of Health regulations, informed consent was not required as all identifying information had been removed prior to data analysis.

### Statistical analysis

The comparisons of baseline characteristics between Arabs and Jews were carried out using the Student’s t-test for continuous variables and the Chi square test for discrete variables. Age and sex data were complete. The proportions of missing data for the MetS variables were: BMI 17.3%, and 9.8%, high-density lipoprotein cholesterol (HDL-C) 19.2% and 15.8%, triglycerides 18.1% and 14.9% and blood pressure 10.9% and 4.3% for Jews and Arabs, respectively (Comparisons between participants with and without available information for the metabolic syndrome components are in supplementary S1–S5 Tables). The 2007 prevalence rates and weighted four-year incidence rates of diabetes were calculated, employing the direct standardization method to adjust for differences in age and sex distributions, using the overall Jewish and Arab populations of Israel for those years (2007 for prevalence and 2008–11 for incidence) as the reference population group. Age-specific rates used for age standardization were in 10-year strata (e.g. 20–29 etc.) [11]. The formula for the calculation of the weighted incidence rates is described in S1 File.

The Cox proportional hazard model was used to study the association between ethnicity and diabetes risk, using age as the time scale. Because the risk of diabetes increases with age, and in order to preserve the proportional hazards assumption, a variable classifying the cohort into 3 categories according to the person’s age on 1.1.2008 (20–49 years, 50–59 years, ≥60 years), was included in the model. The hazard ratios [HRs] for the ethnic groups were adjusted for sex alone or together with the first values recorded of the specific MetS components: triglycerides, BMI, HDL-C and systolic blood pressure (SBP), which were included in the models grouped in quartiles. An extra category was included for missing values for each of the individual MetS components. Since people who have frequent encounters with the health system are also more likely to have blood tests, all analyses were adjusted for the number of visits to the primary care clinic during the year preceding the study period. Interactions of each of the MetS components with ethnic group were analyzed. We compared the average BMI values between people with and without diabetes according to age and ethnic-specific subgroups, using ANOVA and t-test statistics. We also studied the association between diabetes and first BMI recorded before incident diabetes during the study period.

## Results

Selected characteristics of the two groups are shown in Table 1. The total study population included 17,044 Arabs (49% males; mean age 39.4±17.3) and 16,012 Jews (50% males; mean age 40.5 ±17.6). The Arab population had significantly higher BMI (27.7±5.7) than the Jewish population (26.0±5.3). Other variables (HDL-C, triglycerides, SBP and diastolic blood pressure [DBP]), were also significantly worse among the Arab cohort than the Jewish cohort (Table 1).

**Table 1:** Baseline characteristics of the study cohort.

	Jews n = 16,012	Arabs n = 17,044	P
**Age, years, mean ± SD**	40.5 ±17.6	39.4±17.3	< 0.0001
**Males, N (%)**	50	49	< 0.0001
**BMI, kg/m**^**2**^**, mean ± SD**	26.0± 5.3	27.7±5.7	< 0.0001
**HDL-C, mg/dL, mean ± SD** **Men** **Women**	43±10 54 ± 13	41±9 50 ±11	< 0.0001
**Triglycerides, mg/dL,** **median (IQR)**	105, (75,149)	110 (77, 160)	0.02
**SBP, mmHg, mean ± SD**	120±14	121±14	< 0.0001
**DBP, mmHg, mean ± SD**	73± 8	75±7	< 0.0001

Abbreviations: BMI: body mass index; HDL-C: HDL cholesterol; IQR—interquartile range; SBP: systolic blood pressure; DBP: diastolic blood pressure

### Diabetes incidence and prevalence

The age and sex-standardized diabetes cumulative 4-year incidence rates per 100 persons (95% confidence interval [CI] in parentheses) were higher among Arabs 2.9 (2.7–3.1) than among Jews 1.8 (1.7–1.9(, and were evident in all age groups (Table 2). While diabetes incidence rates were higher among Jewish males than among Jewish females, no similar difference was observed among Arab males and females (Table 2).

**Table 2:** Standardized and age-specific prevalence and weighted cumulative 4-yr incidence per 100 persons of diabetes (95% confidence interval)*.

	All		Male		Female	
Age (years)	Jewish-Israelis	Arab-Israelis	Jewish-Israelis	Arab-Israelis	Jewish-Israelis	Arab-Israelis
**All ages** **Prevalence** **Incidence**	10.3 (9.7,10.9) 1.8 (1.7,1.9)	18.4 (17.6, 19.1) 2.9 (2.7, 3.1)	10.7 (9.9,11.6) 2.0 (1.8,2.2)	16.7 (15.7, 17.8) 2.7 (2.5, 3.0)	9.7 (8.9,10.6) 1.6 (1.5, 1.8)	20.0 (19.0, 21.2) 3.0 (2.7, 3.2)
**20–49** **Prevalence** **Incidence**	2.7 (2.3, 3.0) 0.5 (0.4,0.6)	5.7 (5.3, 6.2) 1.1 (1.0, 1.2)	2.7 (2.3, 3.2) 0.6 (0.5, 0.7)	5.2 (4.6, 5.9) 1.1 [0.9, 1.2)	2.6 (2.1, 3.1) 0.4 (0.3,0.5)	6.2 (5.6, 7.0) 1.1 (0.9, 1.2)
**50–59** **Prevalence** **Incidence**	15.0 (13.2,16.9) 2.6 (2.2, 3.0)	32.8 (30.1, 35.8) 4.3 (3.8, 4.8)	17.6 (15.0,20.6) 2.8 (2.2,3.4)	32.9 (28.9, 37.3) 4.0 (3.2, 4.7)	12.4 (10.2,14.8) 2.4 (1.9, 2.9)	32.8 (29.0, 36.8) 4.6 (3.9, 5.4)
**60 +** **Prevalence** **Incidence**	29.8 (27.7,31.4) 4.6 (4.1, 5.0)	45.6 (43.0, 48.2) 7.1 (6.4, 7.9)	32.4 (29.3, 35.7) 5.4 (4.6, 6.1)	42.7 (39.2, 46.5) 7.3 (6.3, 8.2)	27.2 (24.5,30.2) 3.9 (3.3, 4.4)	48.3 (44.6, 52.1) 6.8 (5.8, 7.7)

Age-specific prevalence and incidence rates were calculated for 10 years intervals (e.g. 20–29, etc.) and then grouped into 3 categories according to the person’s age at the start of follow up.

*All estimates were weighted by the survey sample weight to allow for estimates to be generalizable to the overall Jewish and Arab population of Israel.

Arabs had a much higher overall age- and sex-adjusted diabetes prevalence than Jews; 18.4 (17.6–19.1) and 10.3 (9.7–10.9), respectively. This ethnic difference was observed in all age- and sex-specific subgroups (Table 2). Unlike the sex differences in diabetes incidence, the prevalence of diabetes did not differ by sex among Jews. However, Arab females had a much higher prevalence of diabetes than Arab males; 20.0 (19.0–21.2) vs. 16.7 (15.7–17.8), respectively. This female preponderance was observed mainly among Arabs ≥ 60 years old (Table 2).

The HRs for diabetes among younger Arabs (age 20–49 years) were 2.04 (95% CI, 1.74–2.40) and 1.56 (95% CI, 1.34–1.81) in older participants (age ≥ 60 years), (Table 3). Adjustment for the MetS components mildly attenuated the HR estimates associated with Arab ethnicity across all age subgroups, mainly in those <50 years where BMI adjustment changed HR from 2.04 (95% CI, 1.74–2.40) to 1.64 (95% CI, 1.40–1.92) (Table 3).

**Table 3:** Cox proportional hazard ratios (95% confidence interval) for incident diabetes by ethnicity, age, sex and components of the metabolic syndrome*.

**Age** **(years)**	**Model** **adjusted for:**	**Sex**	**sex and triglycerides**	**sex and** **BMI**	**sex and** **HDL-C**	**sex and** **SBP**
20–49	Jews (reference), n = 11244	1.0	1.0	1.0	1.0	1.0
	Arabs n = 12145	2.04 (1.74–2.40)	1.90 (1.62–2.22)	1.64 (1.40–1.92)	1.86 (1.59–2.18)	1.90 (1.62–2.22)
50–59	Jews (reference) n = 1533	1.0	1.0	1.0	1.0	1.0
	Arabs n = 1079	1.56 (1.27–1.92)	1.49 (1.21–1.84)	1.35 (1.09–1.66)	1.43 (1.16–1.76)	1.50 (1.21–1.84)
60 and up	Jews (reference) n = 1906 Arabs n = 1459	1.0 1.56 (1.34–1.81)	1.0 1.52 (1.31–1.77)	1.0 1.40 (1.21–1.64)	1.0 1.51 (1.30–1.76)	1.0 1.54 (1.32–1.79)

* Table includes data on subjects not diagnosed with diabetes until 1/1/2008.

All analyses were adjusted to the number of visits during 2007.

Abbreviations: BMI: body mass index; HDL-C: high density lipoprotein cholesterol; SBP: systolic blood pressure

### BMI according to ethnicity and diabetes status

Across all age groups and for both Arabs and Jews, subjects who developed diabetes during follow-up had on average a higher baseline BMI than subjects who remained non-diabetic (Table 4). Non-diabetic Arabs had a significantly higher BMI than non-diabetic Jews (p<0.0001). However, this was mainly due to higher mean BMI values in females. Arab females who developed diabetes had higher baseline BMI values than Jewish females who developed diabetes during follow-up. No such ethnic difference was observed among males (Table 4).

**Table 4:** BMI among patients with incident diabetes and healthy controls by ethnicity and age*.

Population	<50			50–59			60+		
All	Non-diabetic	Diabetic	p-value	Non-diabetic	Diabetic	p-value	Non-diabetic	Diabetic	p-value
Jews	(n = 8892) 24.97 ± 4.99	(n = 178) 30.36 ± 6.17	<0.0001	(n = 1147) 27.17 ± 5.08	(n = 130) 29.4 ± 5.82	<0.0001	(n = 1348) 27.1 ± 4.7	(n = 255) 29.1 ± 5.2	<0.0001
Arabs	(n = 10327) 26.35 ± 5.04	(n = 439) 31.53 ± 6.47	<0.0001	(n = 769) 29.5 ± 5.29	(n = 153) 32.04 ± 5.5	<0.0001	(n = 993) 28.4 ± 5.4	(n = 319) 29.5 ± 5.6	0.002
p-value	<0.0001	0.039		<0.0001	0.0001		<0.0001	0.6	
Female									
Jews	(n = 4599) 24.6±5.4	(n = 86) 30.9±7.1	<0.0001	(n = 626) 27.1±5.7	(n = 61) 30.0±6.5	0.0002	(n = 718) 27.6±5.2	(n = 116) 30.3±5.8	<0.0001
Arabs	(n = 5407) 26.6±5.6	(n = 229) 32.8±6.8	<0.0001	(n = 419) 31.0±5.5	(n = 90) 33.9±5.8	<0.0001	(n = 490) 30.2±6.0	(n = 145) 31.6±6.1	0.018
p-value	<0.0001	0.03		<0.0001	0.0001		<0.0001	0.08	
Male									
Jews	(n = 4293) 25.4±4.4	(n = 92) 29.8±5.2	<0.0001	(n = 521) 27.2±4.2	(n = 69) 28.8±5.1	0.01	(n = 630) 26.5±3.9	(n = 139) 28.1±4.5	0.0001
Arabs	(n = 4920) 26.1±4.3	(n = 210) 30.1±5.8	<0.0001	(n = 350) 27.7±4.3	(n = 63) 29.4±3.8	0.004	(n = 503) 26.7±4.0	(n = 174) 27.8±4.5	0.004
p-value	<0.0001	0.6		0.1	0.5		0.4	0.6	

*The first BMI recorded during the study period was used for this analysis. Diabetic participants whose BMI values were missing or whose first BMI value was recorded after developing diabetes were excluded; thus BMI was available for 81.4% Jews and 88.5% Arabs. BMI results are presented as mean ±SD.

In rows- statistically significant difference in comparisons within age and diabetes status (Yes/No) subgroups. In columns- statistically significant difference in comparisons between and Jews versus Arabs subgroups. Abbreviations: BMI: body mass index

## Discussion

This study provides data on the age and sex specific prevalence and incidence rates of diabetes in a large population-based cohort among Arabs and Jews in Israel. We found that the prevalence of diabetes among Arabs in Israel is high, affecting 18.4% of all participants over 20 years of age and almost half of the people 60 years and older. A much lower diabetes prevalence was found among Jews; 10.3% over 20 years of age and 29.8% in subjects that were 60 years and older. Reports from other urban indigenous adult Arab populations in the Middle East indicate increasing rates of diabetes; 7.2% in Egypt [12], 22.9% in Jordan [13] to 21.9% in Saudi Arabia [12]. However, there is enormous diversity in diabetes rates among communities within each one of these countries. [12, 14].

In our study, diabetes was more prevalent in Arab females than in Arab males, 20% and 16.7%, respectively. No such sex difference was found among the Jews. Our results with regards to Arabs differ from another Israeli study, where sex difference in diabetes prevalence did not reach statistical significance [7]. A partial explanation for this may be from the differences in the study methodology and the much larger cohort size in the present study.

In Egypt, Saudi Arabia and several other Arab countries in the region, no difference between sexes was noted, [12, 15] while among urban Arab immigrants in the United States, the prevalence rate of diabetes was lower among women than among men; 15.5% and 20.1%, respectively [16–17].

Prevalence data are affected by disease incidence and overall survival. A recent study found that diabetes explain 20% of the excess mortality among Arabs compared to Jews >45 years of age in Israel [8]. To decipher the relative role of each factor, we looked at incidence data. In our study, an increase in incident diabetes was evident among Arabs in all age groups. Among the younger Arab Israelis (<50 years) the HRs for diabetes was double in comparison to Jews. BMI seems to play a role, as Arabs and mainly Arab females up to the age of 60 years, had higher mean BMI values and adjustment for this factor did attenuate some of the overall risk. Nevertheless, Arab ethnicity alone remained a risk factor for diabetes, even after adjusting for triglycerides, HDL-C and systolic blood pressure, it seem to decline with age. Aging per se is accompanied with increased risk for diabetes, thus masking the ethnicity effect between Arabs and Jews. It also may be the result of a smaller gap in modifiable lifestyle factors in the older subjects, both Arabs and Jews. The paramount role of intensive lifestyle intervention in preventing incident diabetes among elderly participants and in reducing ethnic differences in diabetes incidence among Whites, Blacks, Hispanics, and Asian populations, was documented in the Diabetes Prevention Program Outcomes Study and in the Women’s Health Study [18, 19].

### Causes of ethnic differences

It is evident that significant differences exist between Arabs and Jews for both diabetes prevalence and incidence, especially among younger people. However, it is not clear whether these differences arise from underlying genetic susceptibility, dietary differences, income disparity, education, social status or acculturation differences to modern lifestyle. Dietary patterns do seem to differ among Arabs and Jews living in the same geographic region, with Arabs maintaining a high fat, high refined cereal diet, as compared to Jews who consume healthier foods [4]. This trend has also been seen in Arab American immigrants where 70% of the study population followed an ethnically traditional diet [20]. Lack of acculturation to a modern western lifestyle may also contribute to this difference, as seen in Arab American immigrants where this lack was significantly correlated with diabetes prevalence [17]. However, it is not clear whether this is caused by failure to adopt preventative measures such as a healthy diet, exercise and appropriate BMI, or the mitigating factors of socioeconomic status, subjective socioeconomic status, social isolation and exclusion [21, 22]. Across the Middle Eastern world, diabetes prevalence is increasing rapidly, with patient numbers expected to reach epidemic proportions by 2030, if no interventions are successful. Increased wealth in many of these countries has caused rapid changes in dietary habits, consisting of higher fat intake with a more sedentary lifestyle, increase in service sector jobs and decline in outdoor activities. Saudi Arabia and Bahrain, for example, are now considered to be within the ten countries with the highest diabetes prevalence [23]. With regard to income, the opposite trend has been noted in developed countries, such as Canada, where diabetes risk is significantly higher in lower compared to higher income groups. This income gap was widest among younger people and females [24]. Another study among the low income West Bank population showed that diabetes risk continues to rise as well as their mean BMIs and lack of physical exercise, whilst other risk factors such as high cholesterol and smoking decreased between 1998 and 2009 [25].

### Limitations

The data in our study was collected for clinical or administrative purposes and is limited to observations made during clinic visits, and thus could theoretically, be biased toward overestimation of the prevalence and incidence rates, because subjects tend to visit medical clinics when they are ill. However, Arab and Jewish residents in Israel have universal access to healthcare, including free access of all participants to family physicians and laboratory testing. According to national health and social surveys conducted in Israel by the Central Bureau of Statistics, the rates of health services utilization in relation to physician visits, blood pressure and blood tests, are high and do not significantly differ by ethnicity [26, 27], thus reducing this potential bias. Subjects with missing information on MetS components were more likely to be younger, more often male, and less likely to be diabetic. Since these trends were found among both Arabs and Jews, we do not expect that the missing information introduced a significant bias in the analyses on the effect of ethnicity on diabetes risk that were controlled for the individual MetS components.The information provided in the records was not detailed enough to identify the type of diabetes and our database did not have information on income, diet or physical activity as mitigating risk factors, but these variables were meticulously analysed in the previous study [7].

### Conclusion

Arabs in Israel have high diabetes prevalence and incidence. The excess risk for diabetes among Arabs appears in all age groups and for both sexes and is only partially explained by the high prevalence of obesity. The relevance of the various social determinants of health (i.e. socioeconomic status, subjective socioeconomic status, isolation and exclusion) on modifiable causes of diabetes (i.e. BMI, physical activity, mental stress etc.) to the increase in diabetes risk in Arabs as well as genetic factors deserves further investigation. Increased awareness and early intervention of the Israeli healthcare system and cooperation with the Arab community is of paramount need.

## Supporting information

S1 Table Body mass index information. Information on BMI was not available for 9.8% of Arab participants and 17.3% of Jewish participants.
(DOCX)

S2 Table Triglycerides information. Information on triglyceride levels was not available for 14.8% of Arab participants and 18.1% of Jewish participants.
(DOCX)

S3 Table HDL-cholesterol information. Information on HDL-C was not available for 15.8% of Arab participants and 19.2% of Jewish participants.
(DOCX)

S4 Table Systolic blood pressure. Information on systolic blood pressure was not available for 4.3% of Arab participants and 10.9% of Jewish participants.
(DOCX)

S5 Table Diastolic blood pressure. Information on diastolic blood pressure was not available for 4.3% of Arab participants and 10.8% of Jewish participants. NA = Not available Total diabetes: prevalent diabetes by 2007, plus cumulative incident diabetes between 2008–2011.
(DOCX)

S1 File Formula for the calculation of the weighted incidence rates. (DOCX)
