# Supplementary material for: Adult Arabs have higher risk for diabetes mellitus than Jews in Israel
Source: PLoS One. 2017 May 8;12(5):e0176661. doi: 10.1371/journal.pone.0176661 (PMC5421762; doi:10.1371/journal.pone.0176661)
Supplement: S1 Table — Information on BMI was not available for 9.8% of Arab participants and 17.3% of Jewish participants. (DOCX) [file pone.0176661.s001.docx]

**S1 Table: Body mass index information**

|  | Arabs | | | Jews | | |  |
| --- | --- | --- | --- | --- | --- | --- | --- |
|  | Available  N=15,379 | NA  N=1,665* | P | Available  N=13,249 | NA  N=2,763 | P | P-value (for NA Arabs vs. Jews) |
| Age | 39.7 + 17.5 | 36.7 + 15.1 | <0.001 | 40.9 + 17.7 | 38.3 + 16.9 | <0.001 | 0.001 |
| Male, n (%) | 7, 306 (47.5) | 929 (55.8) | <0.001 | 6,434  (48.6) | 1,515  (54.8) | <0.001 | 0.53 |
| Total diabetes by 2011 | 3,290 (21.4) | 153 (9.2) | <0.001 | 1,863  (14.1) | 195  (7.1) | <0.001 | 0.01 |

Information on BMI was not available for 9.8% of Arab participants and 17.3% of Jewish participants.
