# Supplementary material for: Adult Arabs have higher risk for diabetes mellitus than Jews in Israel
Source: PLoS One. 2017 May 8;12(5):e0176661. doi: 10.1371/journal.pone.0176661 (PMC5421762; doi:10.1371/journal.pone.0176661)
Supplement: S2 Table — Information on triglyceride levels was not available for 14.8% of Arab participants and 18.1% of Jewish participants. (DOCX) [file pone.0176661.s002.docx]

**S2 Table: Triglycerides information**

|  | Arabs | | | Jews | |  | |
| --- | --- | --- | --- | --- | --- | --- | --- |
|  | Available  N=14,500 | NA  N=2,544 | P | Available  N=13,119 | NA  N=2,893 | P | P-value (for NA Arabs vs. Jews) |
| Age | 41.4 + 17.5 | 28.4 + 10.4 | <0.001 | 42.7 + 18.0 | 30.3 + 11.0 | <0.001 | <0.001 |
| Sex (male) | 6,445 (44.4) | 1,790  (70.4) | <0.001 | 5,972 (45.5) | 1,977 (68.3) | <0.001 | 0.11 |
| Total diabetes by 2011 | 3,401 (23.5) | 42  (1.7) | <0.001 | 2,031 (15.5) | 27 (0.9) | <0.001 | 0.02 |

Information on triglyceride levels was not available for 14.8% of Arab participants and 18.1% of Jewish participants.
