# Supplementary material for: Adult Arabs have higher risk for diabetes mellitus than Jews in Israel
Source: PLoS One. 2017 May 8;12(5):e0176661. doi: 10.1371/journal.pone.0176661 (PMC5421762; doi:10.1371/journal.pone.0176661)
Supplement: S3 Table — Information on HDL-C was not available for 15.8% of Arab participants and 19.2% of Jewish participants. (DOCX) [file pone.0176661.s003.docx]

**S3 Table: HDL-cholesterol information**

|  | Arabs | | | Jews | | |  |
| --- | --- | --- | --- | --- | --- | --- | --- |
|  | Available  N=14,349 | NA  N=2,695 | P | Available  N=12,932 | NA  N=3,080 | P | P-value (for NA Arabs vs. Jews) |
| Age | 41.5 + 17.5 | 28.3 + 10.3 | <0.001 | 42.9 + 18.0 | 30.1 + 10.8 | <0.001 | <0.001 |
| Sex (male) | 6,378  (44.4) | 1,857 (68.9) | <0.001 | 5,898 (45.6) | 2,051 (66.6) | <0.001 | 0.06 |
| Total diabetes by 2011 | 3,400 (23.7) | 43  (1.6) | <0.001 | 2,031 (15.7) | 27  (0.9) | <0.001 | 0.02 |

Information on HDL-C was not available for 15.8% of Arab participants and 19.2% of Jewish participants.
