# Supplementary material for: Adult Arabs have higher risk for diabetes mellitus than Jews in Israel
Source: PLoS One. 2017 May 8;12(5):e0176661. doi: 10.1371/journal.pone.0176661 (PMC5421762; doi:10.1371/journal.pone.0176661)
Supplement: S4 Table — Information on systolic blood pressure was not available for 4.3% of Arab participants and 10.9% of Jewish participants. (DOCX) [file pone.0176661.s004.docx]

**S4 Table: Systolic blood pressure**

|  | Arabs | | | Jews | | |  |
| --- | --- | --- | --- | --- | --- | --- | --- |
|  | Available  N=16,314 | NA  N=730 | P | Available  N=14,266 | NA  N=1,746 | P | P-value (for NA Arabs vs. Jews) |
| Age | 39.9 + 17.4 | 28.5 + 9.5 | <0.001 | 41.2 + 17.7 | 34.4 + 15.6 | <0.001 | <0.001 |
| Sex (male) | 7,769 (47.6) | 466 (63.8) | <0.001 | 6,904 (48.4) | 1,045 (59.9) | <0.001 | 0.07 |
| Total diabetes by 2011 | 3,431 (21.0) | 12  (1.6) | <0.001 | 1,976 (13.9) | 82  (4.7) | <0.001 | <0.001 |

Information on systolic blood pressure was not available for 4.3% of Arab participants and 10.9% of Jewish participants.
