# Supplementary material for: Adult Arabs have higher risk for diabetes mellitus than Jews in Israel
Source: PLoS One. 2017 May 8;12(5):e0176661. doi: 10.1371/journal.pone.0176661 (PMC5421762; doi:10.1371/journal.pone.0176661)
Supplement: S5 Table — Information on diastolic blood pressure was not available for 4.3% of Arab participants and 10.8% of Jewish participants. NA = Not available Total diabetes: prevalent diabetes by 2007, plus cumulative incident diabetes between 2008–2011. (DOCX) [file pone.0176661.s005.docx]

**S5 Table: Diastolic blood pressure**

|  | Arabs | | | Jews | | |  |
| --- | --- | --- | --- | --- | --- | --- | --- |
|  | Available  N=16,310 | NA  N=734 | P | Available  N=14,274 | NA  N=1,738 | P | P-value (for NA Arabs vs. Jews) |
| Age | 39.9 + 17.4 | 28.7 + 9.7 | <0.001 | 41.2 + 17.7 | 34.4 + 15.6 | <0.001 | <0.001 |
| Sex (male) | 7,765 (47.6) | 470 (64.0) | <0.001 | 6,905 (48.4) | 1,044 (60.1) | <0.001 | 0.07 |
| Cumulative diabetes incidence by 2011 | 3,428 (21.0) | 15  (2.0) | <0.001 | 1,975 (13.8) | 83  (4.8) | <0.001 | <0.001 |

Information on diastolic blood pressure was not available for 4.3% of Arab participants and 10.8% of Jewish participants. NA=Not available
Total diabetes: prevalent diabetes by 2007, plus cumulative incident diabetes between 2008-2011.
