# Supplementary material for: Adult Arabs have higher risk for diabetes mellitus than Jews in Israel
Source: PLoS One. 2017 May 8;12(5):e0176661. doi: 10.1371/journal.pone.0176661 (PMC5421762; doi:10.1371/journal.pone.0176661)
Supplement: S1 File — (DOCX) [file pone.0176661.s006.docx]

**S1 File: Formula for the calculation of the weighted incidence rates**

For any year j and m age groups the ASR will be;

$$\sum_{i=1}^{m} \frac{r_{ij}}{n_{ij}}\times\frac{N_{ij}}{N_{j}}$$

And thus the weighted ASR for 4 years and 16 age groups will be;

$$\sum_{i=1}^{16} \left[ \frac{\sum_{j=1}^{4} r_{ij}}{\sum_{j=1}^{4} n_{ij}}\times\frac{\sum_{j=1}^{4} N_{ij}}{\sum_{j=1}^{4} N_{j}} \right]$$

where;$\sum_{j=1}^{4} r_{ij}=r_{i}$

$\sum_{j=1}^{4} n_{ij}=n_{i}$

and where the variance is given by;

$$\sum_{i=1}^{16} \left[ \frac{r_{i}\left( n_{i} \right.-\left. r_{i} \right)}{n_{i}^{3}}\times\left\{ \frac{\sum_{j=1}^{4} N_{ij}}{\sum_{j=1}^{4} N_{j}} \right\}^{2} \right]$$
